# Expanding laparoscopic pancreaticoduodenectomy to pancreatic-head and periampullary malignancy: major findings based on systematic review and meta-analysis

**DOI:** 10.1186/s12876-018-0830-y

**Published:** 2018-07-03

**Authors:** Ke Chen, Xiao-long Liu, Yu Pan, Hendi Maher, Xian-fa Wang

**Affiliations:** 1 Department of General Surgery, Sir Run Run Shaw Hospital, School of Medicine, Zhejiang University, 3 East Qingchun Road, Hangzhou, 310016 Zhejiang Province China; 2 School of Medicine, Zhejiang University, 866 Yuhangtang Road, Hangzhou, 310058 Zhejiang Province China

**Keywords:** Laparoscopy, Pancreaticoduodenectomy, Adenocarcinoma, Morbidity, Meta-analysis

## Abstract

**Background:**

Laparoscopic pancreaticoduodenectomy (LPD) remains to be established as a safe and effective alternative to open pancreaticoduodenectomy (OPD) for pancreatic-head and periampullary malignancy. The purpose of this meta-analysis was to compare LPD with OPD for these malignancies regarding short-term surgical and long-term survival outcomes.

**Methods:**

A literature search was conducted before March 2018 to identify comparative studies in regard to outcomes of both LPD and OPD for the treatment of pancreatic-head and periampullary malignancies. Morbidity, postoperative pancreatic fistula (POPF), mortality, operative time, estimated blood loss, hospitalization, retrieved lymph nodes, and survival outcomes were compared.

**Results:**

Among eleven identified studies, 1196 underwent LPD, and 8247 were operated through OPD. The pooled data showed that LPD was associated with less morbidity (OR = 0.57, 95%CI: 0.41~ 0.78, *P* < 0.01), less blood loss (WMD = − 372.96 ml, 95% CI, − 507.83~ − 238.09 ml, *P* < 0.01), shorter hospital stays (WMD = − 197.49 ml, 95% CI, − 304.62~ − 90.37 ml, *P* < 0.01), and comparable POPF (OR = 0.85, 95%CI: 0.59~ 1.24, *P* = 0.40), and overall survival (HR = 1.03, 95%CI: 0.93~ 1.14, *P* = 0.54) compared to OPD. Operative time was longer in LPD (WMD = 87.68 min; 95%CI: 27.05~ 148.32, *P* < 0.01), whereas R0 rate tended to be higher in LPD (OR = 1.17; 95%CI: 1.00~ 1.37, *P* = 0.05) and there tended to be more retrieved lymph nodes in LPD (WMD = 1.15, 95%CI: -0.16~ 2.47, *P* = 0.08), but these differences failed to reach statistical significance.

**Conclusions:**

LPD can be performed as safe and effective as OPD for pancreatic-head and periampullary malignancy with respect to both surgical and oncological outcomes. LPD is associated with less intraoperative blood loss and postoperative morbidity and may serve as a promising alternative to OPD in selected individuals in the future.

## Background

Pancreatic-head and periampullary malignancy mainly include pancreatic duct adenocarcinoma (PDAC) and periampullary adenocarcinoma (PAAC). PDAC causes a considerable amount of cancer-related death worldwide. In fact, PDAC is the fourth deadliest malignancy in developed countries and is predicted to become the second one within several years [1, 2]. PAAC, defined as malignancy located in the distal common bile duct, ampulla of Vater or adjacent duodenum, are uncommon cancers compared to PDAC. However, despite its relatively higher resectability rates compared with PDAC, the long-term survival outcomes of PAAC patients are poor [3]. These cancers represent great challenges for healthcare providers and require a multidisciplinary approach in which pancreaticoduodenectomy (PD) with lymphadenectomy remains the primary curative treatment for patients without distant metastasis.

Minimally invasive surgery, typically characterized by laparoscopic approach, is one of the main surgical advances in the twenty-first Century [4]. It has been applied to complex pancreatic procedures including laparoscopic PD (LPD) for neoplasms on the pancreatic head and periampullar region or laparoscopic distal pancreatectomy (LDP) for these on the pancreatic body or tail. LDP represents a technique, which is technically less demanding, without any reconstruction, whereas LPD is a demanding procedure, which should be performed only in referral centers by experienced surgeons. Now, several meta-analyses have been published regarding LDP for malignancy treatment [5, 6], there was still no meta-analysis on the potential advantages and disadvantages of LPD for cancers. This meta-analysis proposed to dig deeper into the surgical and oncologic outcomes of patients who suffered from pancreatic-head and periampullary malignancy and underwent LPD versus open PD (OPD).

## Methods

### Study selection

We systematically searched the relevant literature using PubMed, Embase, Cochrane Library, and EBSCO for articles published up to March 2018 The search terms “minimally invasive”, “laparoscopy”, “Whipple”, “pancreaticoduodenectomy”, “pancreatic ductal adenocarcinoma”, “pancreatic cancer”, “ampullary cancer”, “ampulla of Vater”, and “periampullary neoplasm” were utilized. All eligible studies were retrieved, and their bibliographies were further checked for other potential publications. The language of the publications was confined to English.

### Inclusion criteria

Publications included in this study were based on the following criteria: (1) compared LPD and OPD in patients suffering from pancreatic-head and periampullary malignancy (PDAC and PAAC); (2) reported on at least one of the outcome measures mentioned below; and (3) if there was overlap between authors and/or institutions, the higher quality or more recent publication was selected.

### Data extraction

Information was independently extracted from eligible studies by two authors (Chen K and Liu XL). The following information was collected: ① Primary outcomes: morbidity, mortality, postoperative pancreatic fistula (POPF), margin status, retrieved lymph nodes (RLNs), and long-term survival. ② Secondary outcomes: operative time, intraoperative blood loss, transfusion, and length of hospital stay. The postoperative morbidity was classified according to the Clavien-Dindo classification when possible [7]. POPF were diagnosed in accordance with the International Study Group for Pancreatic Fistula (ISGPF) criteria [8]. Clinically significant POPF was defined as ISGPF grade B/C. Resection margins were considered negative (R0) when no tumor was evident along the transection surface [9].

### Quality assessment

The Newcastle-Ottawa Quality Assessment Scale (NOS) was used to evaluate the quality of non-randomized controlled trials (NRCTs). The quality of each study was scored by taking into account patient selection, comparability of the groups and assessment of the outcomes. The scale ranges from 0 to 9 stars: studies with a score ≥ 6 could be deemed as methodologically sound. Randomized controlled trials (RCTs) were assessed by the Jadad scale. High quality RCTs get more than 2 out of a maximum possible score of 5.

### Statistical analysis

Statistical analysis was performed using the RevMan 5.1 software (Cochrane Collaboration, Oxford, UK). The odds ratio (OR) or weighted mean differences (WMD) with 95% confidence intervals (CI) were calculated for dichotomous and continuous results, respectively. Medians were converted to means by the method introduced by Hozo et al. [10]. The hazard ratio (HR) was used as a summary statistic for long-term survival. The log HR and its standard error (SE) were analyzed directly if the studies reported the HR and 95% CI. Otherwise, the log HR and its SE were estimated using the method described by Tierney et al. [11]. The fixed-effect model was firstly used for primary and secondary outcomes, and in case of significant heterogeneity (*I*^*2*^>50% or *Q* test *P* < 0.05) the results were calculated using the random-effect model. Subgroup analysis was carried out according to the tumor primaries (PDAC or PAAC). Funnel plots were used to screen for publication bias based on the R0 rate. The statistical tests were two-sided, and *P* < 0.05 was considered statistically significant.

## Results

### Study characteristics and quality of included studies

**Fig. 1:**
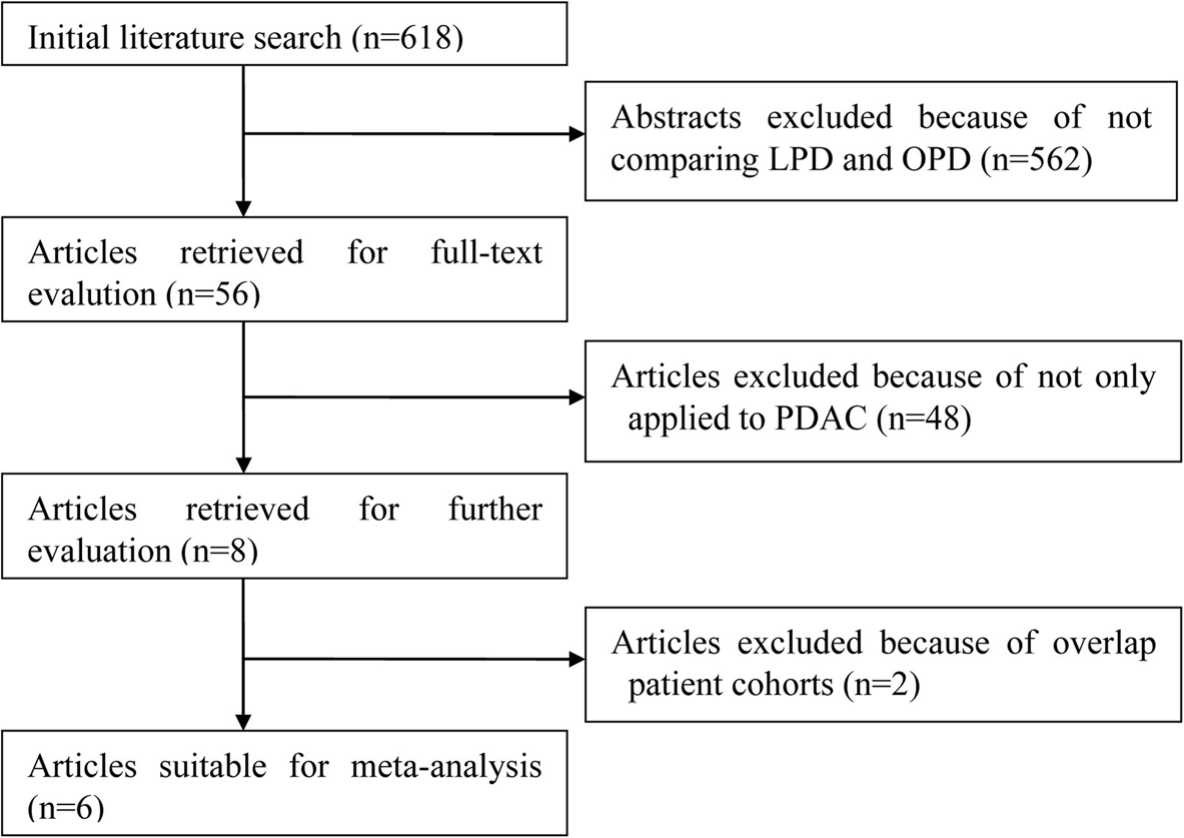
Flow chart of literature search strategies

The search strategy initially generated 668 relevant clinical trials. Of these, articles that did not compare LPD with OPD were excluded based on their titles and abstracts. Thus, 86 articles were selected and a full examination of the text was conducted. A further 73 papers were excluded because the surgical indications of these studies were not restricted to pancreatic-head and periampullary malignancy. Another two publications were then excluded due to overlapping patient cohorts [12, 13]. Finally, a total of 9443 patients [LPD 1196 (12.7%), OPD 8247 (87.3%)] from 11 studies were included [14–24]. Figure 1 illustrates the selection process. Only one RCR was found [22]. The indication of six studies was PDAC [14, 16–20], whereas two studies applied LPD only to PAAC [15, 24], the remaining three researches reported both PDAC and PAAC [21–23]. Table 1 lists the characteristics of these studies and details of the enrolled participants. The RCT conducted by Palanivelu et al. received a Jadad score of 3 [22]. The quality evaluation using NOS showed that the included NRCTs were methodologically sound with four studies receiving 9 stars, five studies receiving 8 stars, and the remaining study receiving 7 stars (Table 1).

**Table 1:** Summary of studies included in the meta-analysis

Author	Region	Design	Year	Study Period	Sample size		Indications	Conversion n (%)	ITT	ISGPF	Clavien–Dindo	Mortality	Quality scores
					LPD	OPD							
Croome	USA	OCS (P,S)	2014	2008–2013	108	214	PDAC	7(6.5)	Yes	Yes	Yes	30d	8
Hakeem	UK	OCS (R,S)	2014	2005–2009	12	12	PAAC	NR	NR	NR	Yes	30d	9
Chen	China	OCS (P,S)	2015	2010–2013	19	38	PDAC	1(5.3)	Yes	Yes	Yes	NR	8
Song	Korea	OCS (R,S)	2015	2007–2012	11	261	PDAC	NR	No	Yes	Yes	30d	8
Dokmak	France	OCS (P,S)	2015	2011–2014	15	14	PDAC	NR	Yes	Yes	Yes	90d	7
Stauffer	USA	OCS (P,S)	2017	1995–2014	58	193	PDAC	14(24.1)	Yes	Yes	Yes	90d	8
Kantor	USA	OCS (R,M)	2017	2010–2013	828	7385	PDAC	E	NR	NR	NR	90d	8
Conrad	USA	OCS (P,S)	2017	2000–2010	40	25	Mixed	9(18.4)^a^	No	Yes	Yes	90d	9
Palanivelu	India	RCT	2017	2013–2015	32	32	Mixed	1(3.1)	Yes	Yes	Yes	90d	3^a^
Khaled	UK	OCS (R,S)	2017	2002–2015	15	15	Mixed	1(6.7)	Yes	Yes	Yes	30d	9
Meng	China	OCS (R,S)	2018	2010–2015	58	58	PAAC	NR	NR	Yes	Yes	30d	9

*OCS* observational clinical study, *RCT* randomized controlled trial, *P* prospectively collected data, *R* retrospectively collected data, *M* muti-centers, *S* single center, *DP* distal pancreatectomy, *PD* pancreatoduodenectomy, *L* laparoscopy, *O* open, *ITT* intention-to-treat analysis, *ISGPF* international study group of pancreatic fistula, *PJ* pancreaticojejunostomy, *DTM* duct-to-mucosa, *E* exclude, *NR* not reported

^a^ Jadad score

### Primary outcomes

**Fig. 2:**
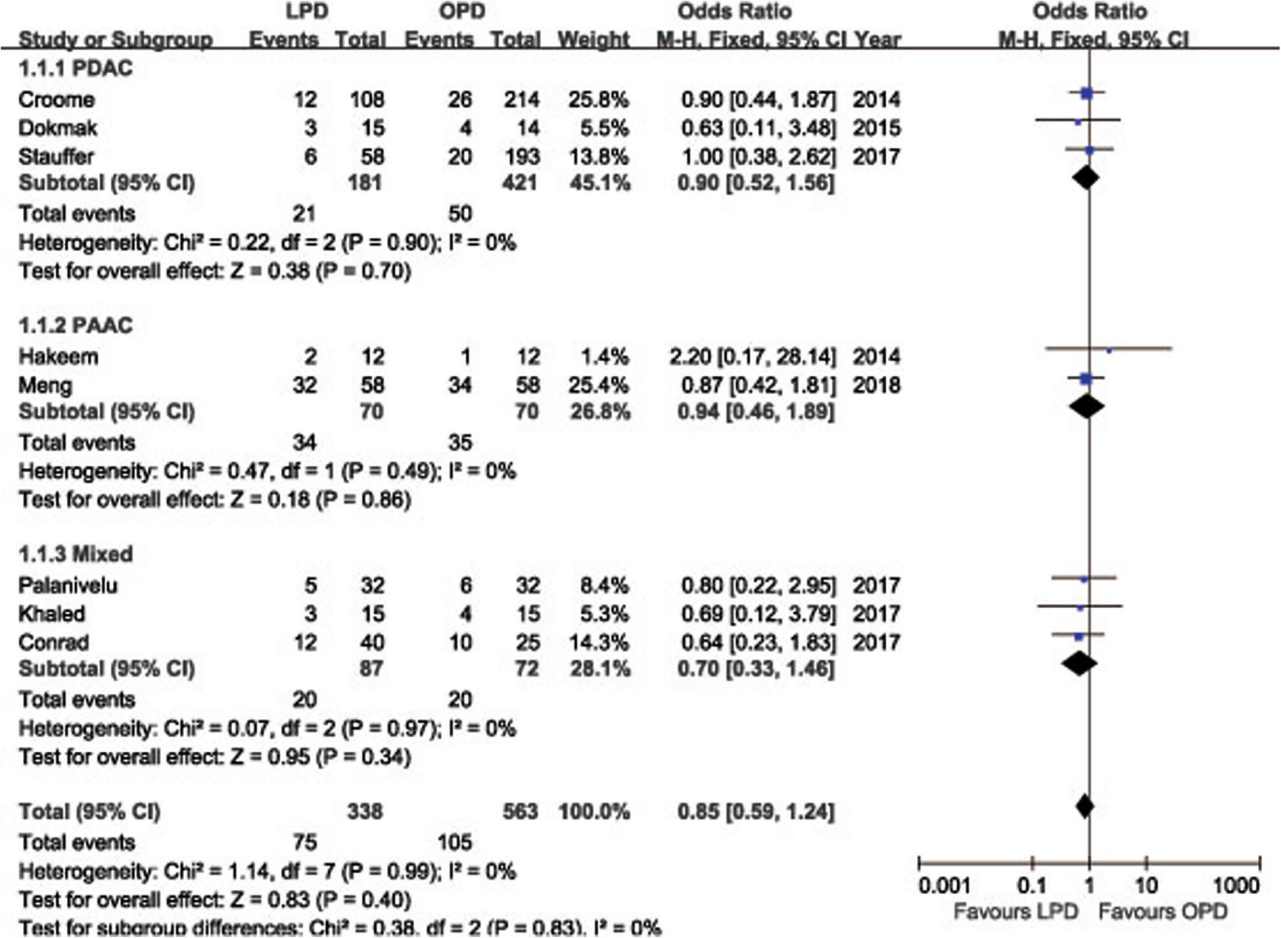
Forest plot of the meta-analysis: overall POPF

**Fig. 3:**
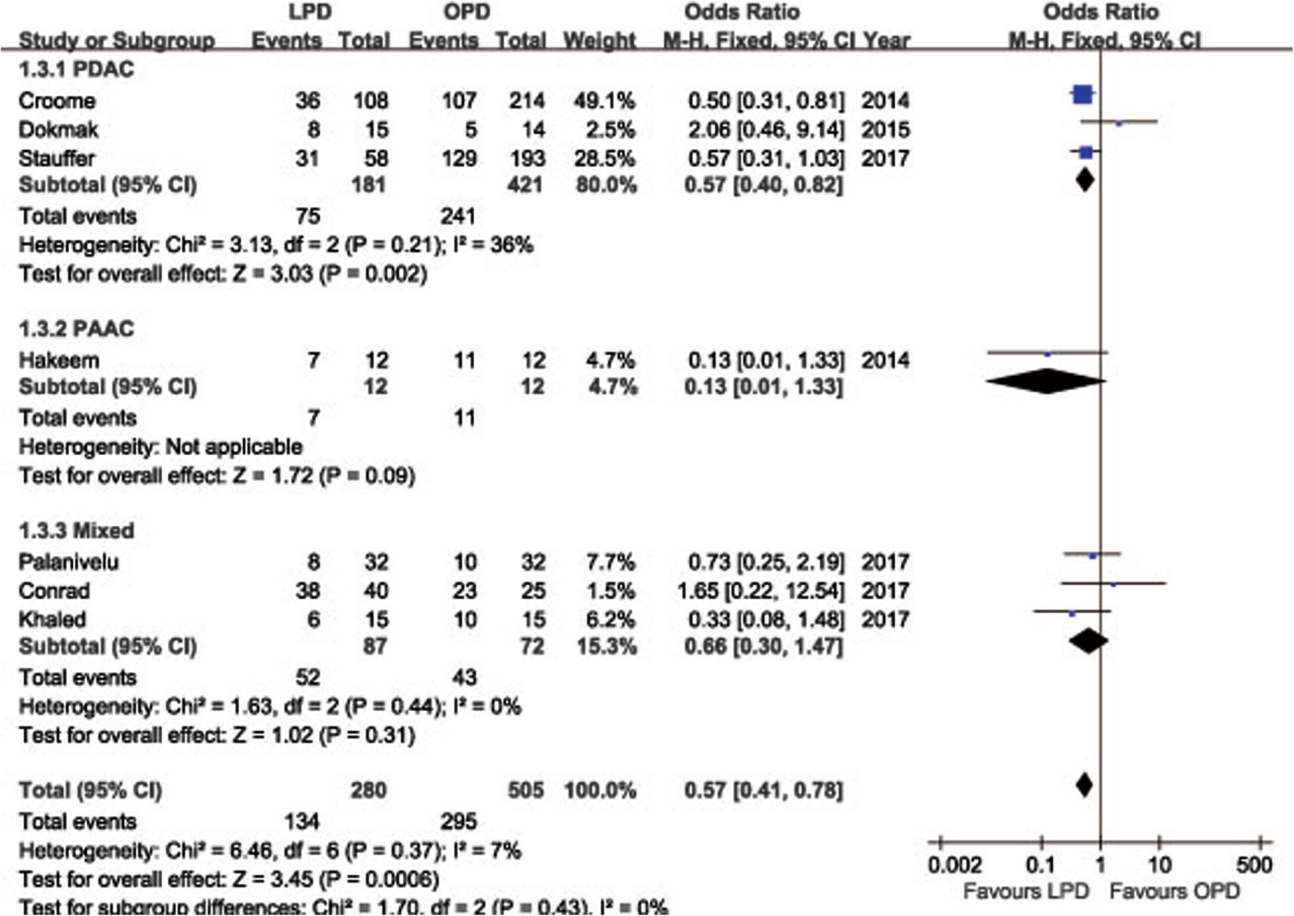
Forest plot of the meta-analysis: morbidity

**Fig. 4:**
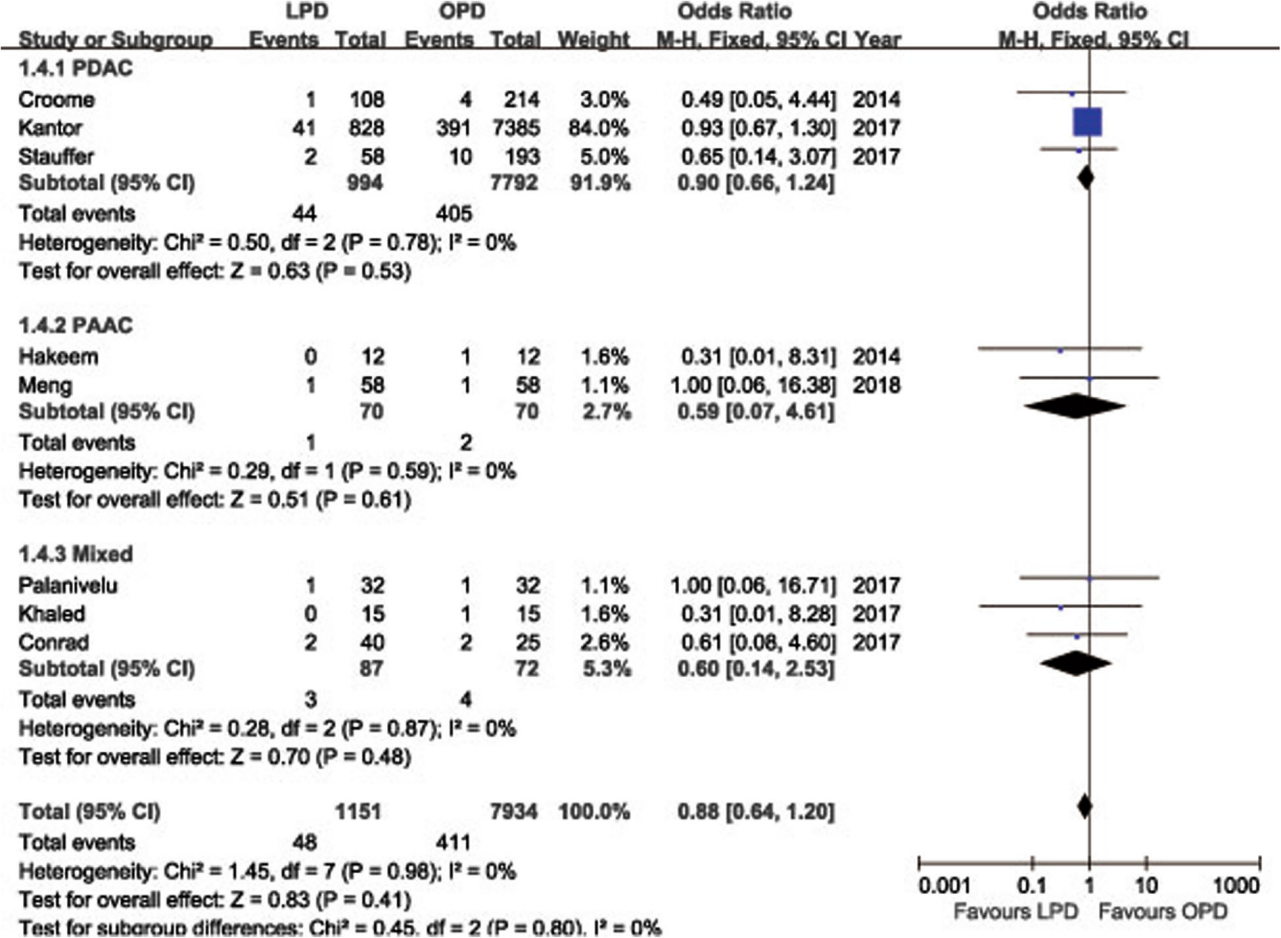
Forest plot of the meta-analysis: mortality

**Fig. 5:**
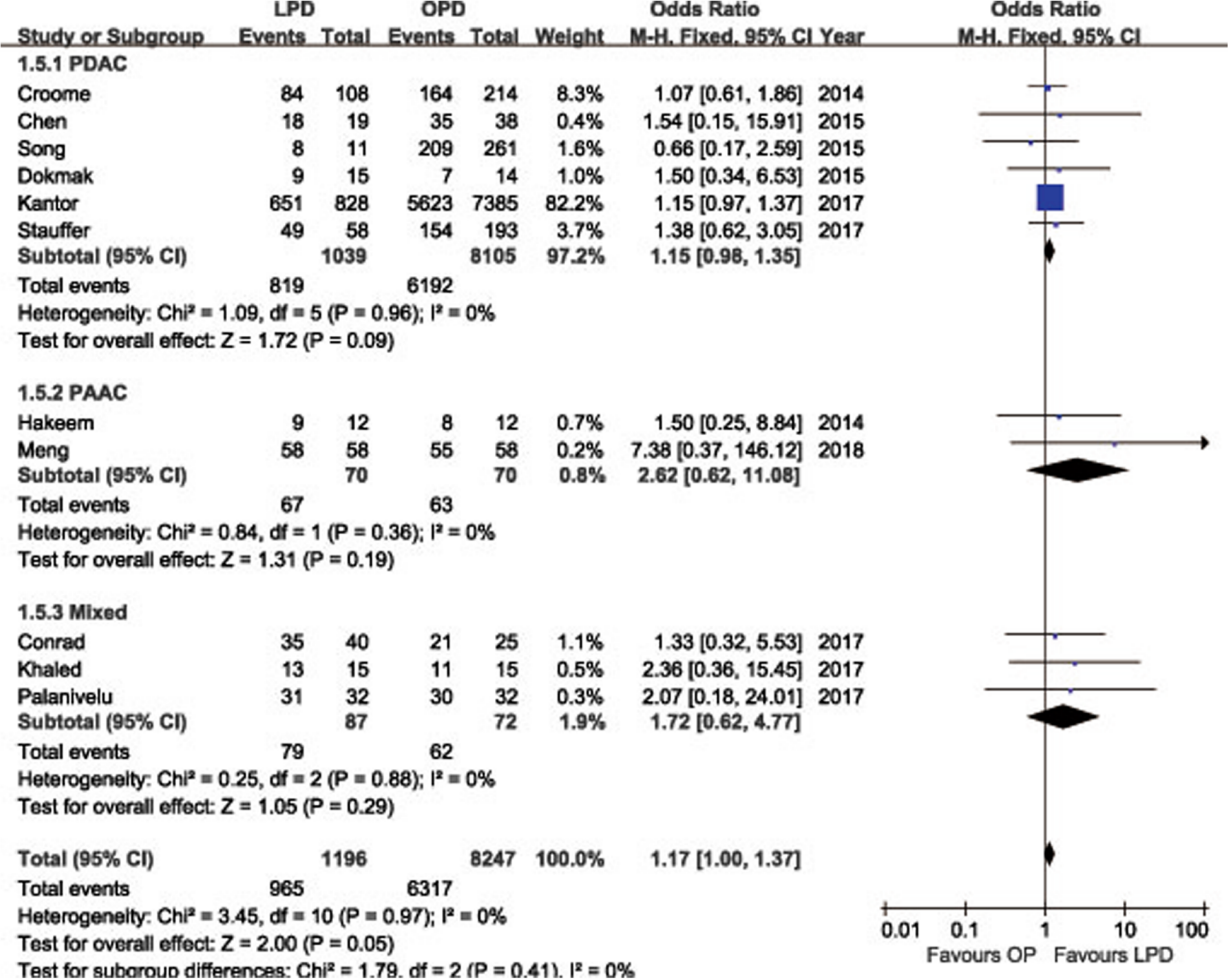
Forest plot of the meta-analysis: R0 rate

**Fig. 6:**
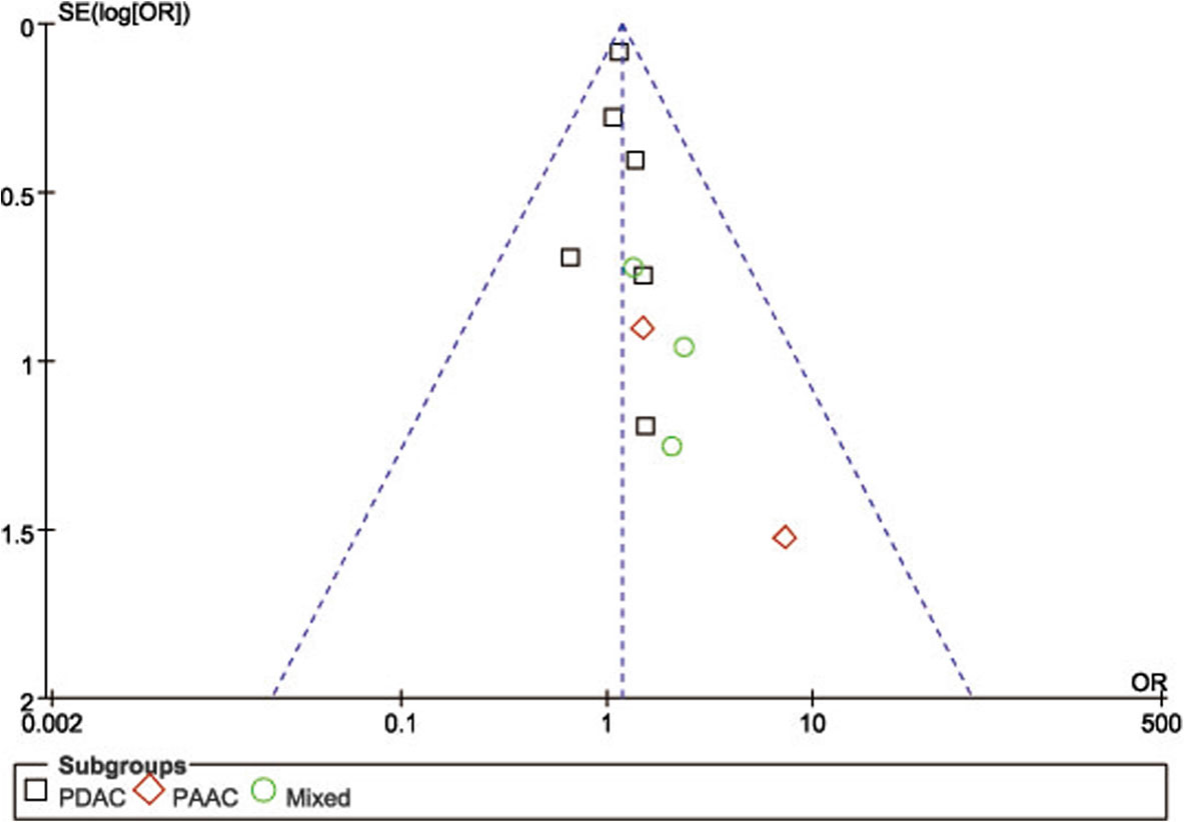
Funnel plots of the R0 rate

**Fig. 7:**
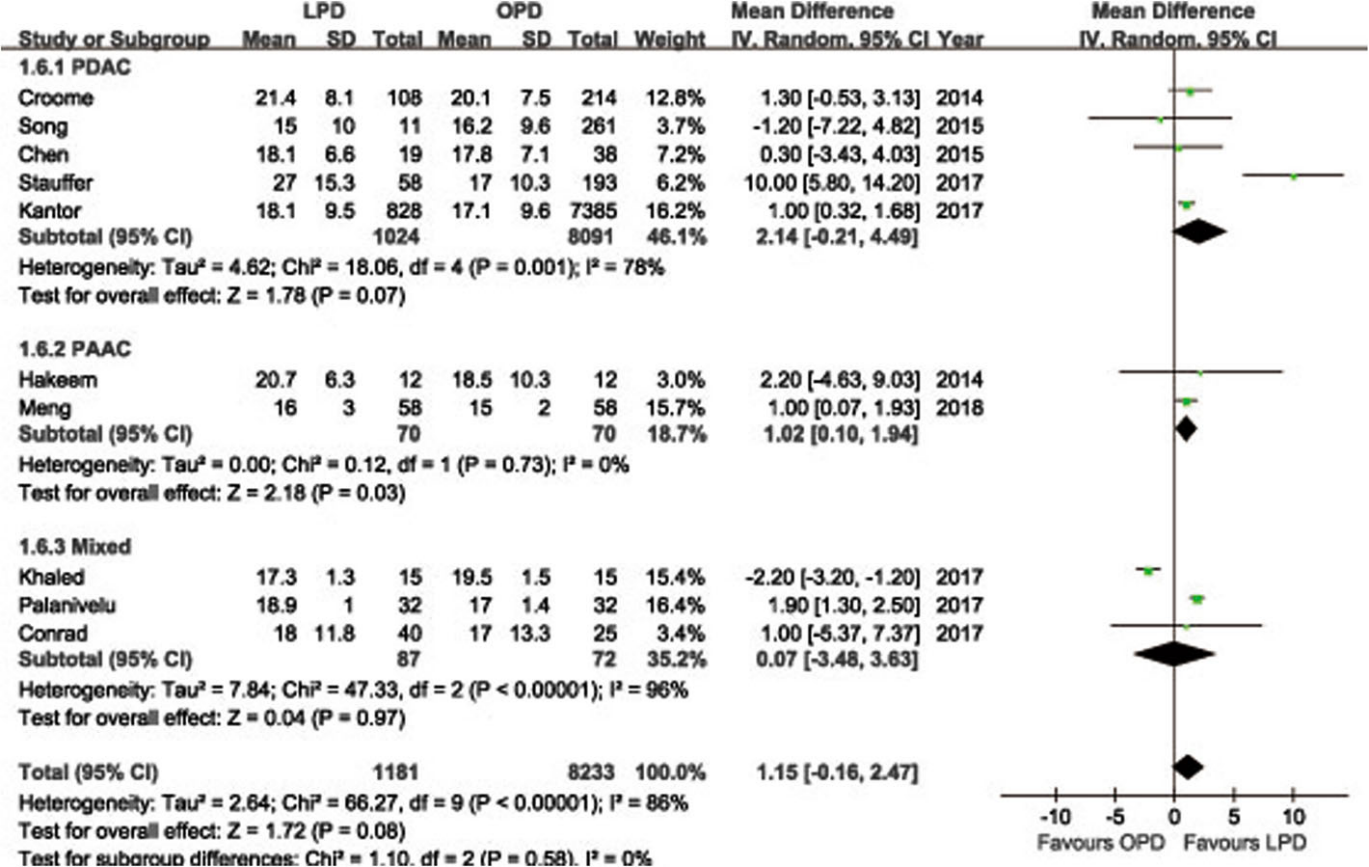
Forest plot of the meta-analysis: retrieved lymph nodes

**Fig. 8:**
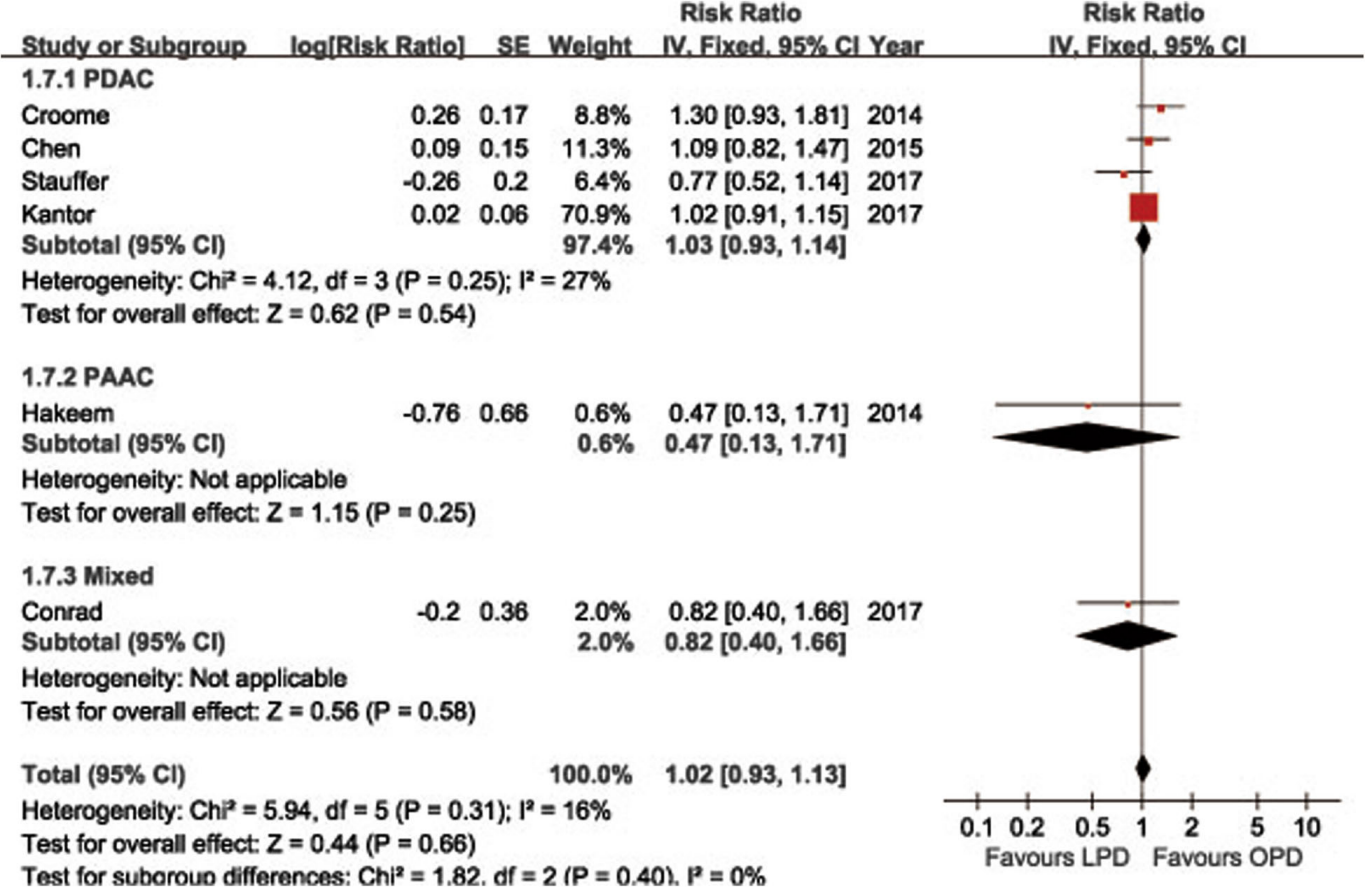
Forest plot of the meta-analysis: overall survival rate

POPF was described in 8 studies [14, 15, 18, 19, 21–24]. Pooling data indicated no significant difference in terms of overall POPF rates between two groups (OR = 0.85, 95%CI: 0.59~ 1.24, *P* = 0.40) (Fig. 2), as well as the clinically significant POPF rates (OR = 0.86, 95%CI: 0.53~ 1.41, *P* = 0.56). The morbidity was available from 7 studies [14, 15, 18, 19, 21–23]. The pooled data indicated that the overall postoperative morbidity was significantly decreased in LPD (OR = 0.57, 95%CI: 0.41~ 0.78, *P* < 0.01) (Fig. 3). Eight studies recorded postoperative mortality [14, 15, 19–24]. Mortality was similar in LPD and OPD for malignancies (OR = 0.88, 95%CI: 0.64~ 1.20, *P* = 0.41) (Fig. 4). Negative margin (R0) rate was reported in all studies. The difference in R0 rate was not significant in pooling results, but had a tendency to be higher in the LPD group than in the OPD group (OR = 1.17, 95%CI: 1.00~ 1.37, *P* = 0.05) (Fig. 5). The funnel plot for studies reporting the ORs of R0 was used to detect publication bias. Visual inspection of the funnel plot revealed symmetry, indicating no serious publication bias, as illustrated in Fig. 6. The RLNs were pooled from 10 studies [14–17, 19–24]. Pooled results revealed a tendency of more RLNs in the LPD group than in the OPD group with a marginal difference (WMD = 1.15, 95%CI: -0.16~ 2.47, *P* = 0.08) (Fig. 7). Six studies reported survival outcomes. The overall survival rate was not found to be different among the two groups (HR = 1.03, 95%CI: 0.93~ 1.14, *P* = 0.54) (Fig. 8). The primary outcomes of the quantitative meta-analysis were summarized in Table 2.

**Table 2:** Results of the meta-analysis

Outcomes	No. Studies	Sample size		Heterogeneity (*P,I*^*2*^)	Model	Overall effect size	95% CI of overall effect	*P*
		LPD	OPD					
Primary Outcomes								
POPF	8	338	563	0.99, 0%	F	OR = 0.85	0.59~ 1.24	0.40
Significant POPF	5	271	512	0.96, 0%	F	OR = 0.86	0.53~ 1.41	0.56
Morbidity	7	280	505	0.37, 7%	F	OR = 0.57	0.41~ 0.78	< 0.01
Mortality	8	1151	7934	0.98, 0%	F	OR = 0.88	0.64~ 1.20	0.41
R0 rate	11	1196	8247	0.97, 0%	F	OR = 1.17	1.00~ 1.37	0.05
Retrieved lymph nodes	10	1181	8233	< 0.01, 86%	R	WMD = 1.15	-0.16~ 2.47	0.08
Overall survival	6	1065	7867	0.31, 16%	R	HR = 1.02	0.93~ 1.13	0.66
Secondary Outcomes								
Operation time (min)	5	271	512	< 0.01, 99%	R	WMD = 87.68	27.05~ 148.32	< 0.01
Blood loss (mL)	5	271	512	< 0.01, 96%	R	WMD = −197.49	− 304.62~ − 90.37	< 0.01
Transfusion	5	296	522	0.36, 7%	F	OR = 0.64	0.50~ 0.84	< 0.01
Hospital stay (days)	9	1166	7948	< 0.01, 85%	R	WMD = −1.07	−3.05~ 0.92	0.29
Tumor size	10	368	862	0.46, 0%	F	WMD = −0.16	−0.31~ − 0.02	0.03

### Secondary outcomes

Five studies reported operative time [14, 19, 22–24]. The operative time in LPD group was longer than that in OPD group (WMD = 87.68 min, 95%CI: 27.05~ 148.32, *P* < 0.01). Also five studies reported blood loss [14, 19, 22–24]. The estimated blood loss was significantly reduced in LPD group (WMD = − 197.49 ml, 95% CI, − 304.62~ − 90.37 ml, *P* < 0.01). A similar result was achieved in the field of blood transfusions (OR = 0.64, 95 %CI: 0.50~ 0.84, *P* < 0.01). Nine studies reported the length of hospital stay [14, 15, 18–24]. The pooled data indicated a comparable length of hospital stay between groups (WMD = − 1.07, 95%CI, − 3.05~ − 0.92, *P* = 0.29). Tumor size was available except in one study [20]. The tumor size of OPD was larger than that of LPD (WMD = − 0.16, 95%CI, − 0.31~ − 0.02, *P* = 0.03). The secondary outcomes of the quantitative meta-analysis are outlined in Table 2.

### Sensitivity analysis

One retrospectively muti-institutional study conducted by Kantor et al. [20], in which only PDAC were included, provided the vast majority of cases. The study offered outcomes of mortality, R0 rate, RLNs, overall survival, and length of hospital stay. We investigated the influence of this study on the overall estimated risk by sequentially removing it from the pooled outcomes. We found only the pooling data of R0 rate changed from marginal difference (OR = 1.17, 95%CI: 1.00~ 1.37, *P* = 0.05) to no significant difference (OR = 1.28, 95%CI: 0.89~ 1.84, *P* = 0.19), whereas the main results presented no obvious changes.

## Discussion

Minimally invasive PD (MIPD), a laparoscopic surgery, was first described by Gagner and Pompin in 1994, and there has been a recent surge of interest for this demanding technique [25, 26]. PD is a complex procedure and the advantages of minimally invasive approaches used to be closely scrutinized. Although several meta-analyses have confirmed the advantages of MIPD over open surgery, there has been no analysis restricted to malignancy. Moreover, effects of oncologic results were not well evaluated due to insufficient data. In Table 3, we present several previous meta-analyses comparing MIPD to OPD for benign and malignant periampullary disease [27–34].

**Table 3:** Previous meta-analyses comparing MIPD with OPD for benign and malignant periampullary disease

Variables	Correa	Nigri	Lei	Qin	de Rooij	Zhang	Peng	Shin^b^	Shin^b^
Year	2014	2014	2014	2014	2016	2016	2017	2017	2017
Included studies	6	8	9	11	19	22	9	8	5
Minimally invasive method	MIPD	MIPD	MIPD	MIPD	MIPD	MIPD	RPD	LPD	RPD
Total MIPD numbers	169	204	209	327	710	1018	245	450	160
POPF	NS	NS	NS	NS	NS	NS	NS	NS	NS
Morbidity	NS	NS	NS	NS^a^	N/A	NS	Favor RPD	NS	NS^a^
Mortality	N/A	NS	NS	NS	NS	NS	NS	N/A	N/A
R0 rate	Favor MIPD	NS	Favor MIPD	NS^a^	Favor MIPD	Favor MIPD	Favor RPD	NS	NS
Retrieved lymph nodes	Favor MIPD	NS^a^	NS	NS	NS	NS^a^	NS	NS	NS
Survival	N/A	N/A	N/A	N/A	N/A	N/A	N/A	N/A	N/A
Operation time	Favor OPD	Favor OPD	Favor OPD	Favor OPD	Favor OPD	Favor OPD	NS	Favor OPD	Favor OPD
Blood loss	Favor MIPD	Favor MIPD	Favor MIPD	Favor MIPD	Favor MIPD	Favor MIPD	N/A	NS	Favor RPD
Transfusion	N/A	NS	Favor MIPD	N/A	N/A	Favor MIPD	N/A	N/A	N/A
Hospital stay (days)	Favor MIPD	Favor MIPD	Favor MIPD	Favor MIPD	Favor MIPD	Favor MIPD	Favor RPD	Favor LPD	Favor RPD

*NS* not significant, *N/A* not available

^a^ not significant but tended to favor MIPD

^b^ one separate study

This meta-analysis selected and summarized the available literature that compared the short-term surgical and long-term survival results of LPD and OPD for malignant periampullary disease. To the best of our knowledge, this is the first meta-analysis comparing LPD versus OPD for the treatment of pancreatic-head and periampullary cancer. No statistically significant differences were identified between the two groups regarding POPF, mortality, overall survival rate, and hospital stay. Operative time was significantly longer in the LPD group. The differences of R0 rate, and RLNs did not reach statistical significance, but tended to be superior in the LPD group. Moreover, LPD exhibited statistically significant benefits in terms of blood loss, and overall postoperative complications.

The progress of LPD was slow due to the threatening complication of POPF. High rates ranging from 4 to 33% for conventional open surgery were previously reported for both benign and malignant lesions [35, 36]. POPF rates range from 11.8 to 55.2% in LPD for malignancy as reported seemed to somewhat higher than the results above (Table 4). It was also reported that LPD for resection of periampullary tumors was associated with higher morbidity, mainly due to severe POPF [18]. However, our pooling data demonstrated comparable rates, regardless of overall POPFs or clinically significant POPFs between LPD and OPD for malignancy. The approaches which could reduce POPFs also can be completed under laparoscope [37]. The end-to-side, duct-to-mucosa pancreaticojejunostomy (PJ) was now the most commonly performed pancreatic anastomosis approach under laparoscopy just as the conventional open approach [38]. Importantly, our results have shown improved overall morbidity of LPD. Since surgical complications, mainly POPFs, were comparable between the two groups because of the same organ and lymphatic resection area of LPD and OPD, the reduced overall complications may be the result of fewer medical complications. PD involves multiple systems and the complexity of performing three anastomoses can result in enormous surgical trauma, which would result in high risks of medical complications. Pulmonary, cardiovascular, and cerebrovascular morbidities were the most frequent systemic complications in major abdominal surgery. Less bleeding and use of transfusion contribute to preserve stable pulmonary and cardiovascular functions [39]. In addition, less pain and earlier ambulation allows patients to restore physiological homeostasis.

**Table 4:** Studies on LPD for pancreatic cancer

	Croome	Hakeem	Chen	Song	Dokmak	Stauffer	Kantor	Conrad	Palanivelu	Khaled	Meng
Age (years)	66.6 ± 9.6	67.0 ± 10.2	–	68.1 ± 7	–	69.9(40.6–84.8)	65.9 ± 10.7	68(45–83)	57.8 ± 2.0	65(35–78)	60.0 ± 9.1
Sex (M/F)	57/51	8/4	–	–	–	32/26	–	26/14	18/14	8/7	32/26
BMI	27.4 ± 5.4	25.8 ± 3.7	–	–	–	25.9(17.7–49.6)	–	23.9(14.9–34.1)	24.9 ± 0.7	23.4(18–26)	22.3 ± 3.0
Tumor size (cm)	3.3 ± 1.0	2.0 ± 1.0	3.0 ± 0.9	2.8 ± 0.6	2.4(1.5–4)	2.5(0.3–10.0)	–	2.5 (0.3–8.0)	3.3 ± 0.7	2.0(0.7–8.0)	1.9(1.5–2.6)
Retrieved LNs	21.4 ± 8.1	20.7 ± 6.3	18.1 ± 6.6	15 ± 10	20(8–59)	27(9–70)	18.1 ± 9.5	18(6–53)	18.9 ± 1.0	18(14–19)	16(15–18)
R0 rate	77.8%	75.0%	94.7%	72.7%	60%	84.5%	79.1%	87.5%	96.9%	86.7%	100%
Operative time (min)	379.4 ± 93.5	–	–	–	–	518(313–761)	–	–	359 ± 14	470(280–660)	475(420–546)
Blood loss (mL)	492.4 ± 519.3	–	–	–	–	250(50–8500)	–	–	250 ± 22	300(50–600)	200(100–325)
POPF	–	16.7%	–	–	20%	11.8%	–	30%	15.6%	20.0%	55.2%
Significant POPF	11%	–	–	–	–	7.8%	–	–	6.3%	20.0%	13.8%
Morbidity	5.6% CD>2	58.3%	–	–	53%	53.4%	–	95%	25.0%	40.0%	15.5% CD>2
Hospital stay (days)	6 (4–118)	14.9 ± 6.6	–	–	15(6–53)	6(4–68)	10.2 ± 8.5	24.5(9–311)	7(5–52)	9.0(7–20)	14.0(11.0–17.3)
Readmission	–	–	–	–	–	22.4% 90d	6.8% 30d	–	6.3% 90d	–	–
Mortality	0.9% 30d	0% 30d	–	0% 30d	0% 90d	3.4% 90d	6.9% 90d	5% 90d	3.1% 90d	0% 30d	1.7% 30d
Survival	MS: 25.3 m	1,3,5y-DFS:100,92,83%; 1,3,5y-OS:100,92,75%.	–	5y-OS: 53.6%	–	1,2,3,4,5y-OS: 66.5,43.3,43.3%, 38.5,32.1%	MS: 20.7 m	MS: 35.5 m; 1,3,5y-DFS: 62.3, 37.9, 25.7%; 1,3,5y-OS: 80.5, 49.2, 39.7%	–	1,3,5y-OS: 100, 80, 67%	MS: 45 m

*OS* overall survival rate, *MS* median survival time, *m* month

The pooling results show that LPD is linked with a tendency of lower positive margin rate (*P* = 0.05) and more retrieved lymph nodes (*P* = 0.08), two of the oncologic outcomes. Appropriate lymphadenectomy is crucial because elimination of a sufficient quantity of lymphadens could help to strengthen the staging accuracy and regional tumor control. In addition, curative R0 resection is referred as the most important factor determining, which is deemed the only chance to survival [40], and the prognostic validity of margin status may be primarily confined to pancreatic head cancers rather than neoplasms in body or tail [41]. Elaborate manipulation and better visualization of critical anatomy could explain our outcomes. However, as our results also indicated a shorter tumor size in LPD group, some researches may include LPD cases of small, easily resectable tumors that would be partial to LPD, the benefit of LPD for margin status and lymph nodes harvesting cannot be confirmed, but at least not inferior to open surgery. Long-term survival rate is crucial the outcome for evaluating surgical interventions in oncological therapy. Periampullary cancer, especially PDAC, has significantly more aggressive inherent tumor biology, for which there are hardly any effect of adjuvant therapy [42, 43]. Large series on PDAC reported 5-year survival rates only around 20% [44, 45], and estimated at only 20–50% for PAAC [3, 46]. None of the previous studies on LPD identify survival advantages of the laparoscopic approach, and our analysis revealed the HR of overall survival rate was comparable between LPD and OPD. We agree with the viewpoint of Croome et al. that neither the laparoscopic or open procedure was technically superior would largely depend on the technique of surgeon and on pathologic analytic variability. Thus, a technically similar oncologic resection could be performed regardless of whether the open or laparoscopic approach was meticulously used [14].

Regarding the operative time and blood loss, our pooled data indicated similar outcomes to the previous studies (Table 3). Because of the intricate dissection and complicated gastrointestinal, LPD present a more demanding and challenging approach for pancreatic surgeons. Kendrick and Cusati report their initial duration of LPD to be approximately 8 h, which improved to 5 h after approximately 50 cases [47]. Therefore, adequate training and optimizing surgical potency to reduce the operative time is required before LPD becomes a generally accepted and sustainable procedure. Another benefit of laparoscopic surgery lies in the enhanced postoperative recovery. Reduced use of analgesic drugs, shortened time of abdominal cavity exposure, and earlier postoperative activities are considered to be the main reasons.

This systematic review and meta-analysis of LDP versus ODP for pancreatic-head and periampullary malignancy represents the most comprehensive collection of evidence available within this field. However, the results should still be explained with caution for several limitations. First, only one study was RCT, while others were NRCTs. Selection biases necessarily consist in surgeons’ or patients’ decision on operation and adjuvant therapy. Moreover, various biases are real concerns because hardly any of the included studies employed standardized appraisal for the end points. Second, clinical heterogeneity needs significant attention. The surgical techniques, take the different the extension of lymph node dissection for example, and histopathological protocols were variable in both open and laparoscopic groups. Third, there are biological differences between PDAC and PAAC. Although a subgroup analysis was conducted, we should still admit various histologies unpowered our outcomes because several studies did not differentiate PDAC and PAAC in their studies, and even in PAAC, there are several different histologies. Last but not least, it should be kept in mind that these studies were on behalf of the best centers’ experience on LPD around the world. The literature showed that specialization in pancreatic surgery results in both better short- and long-term survival [48]. Obtained conclusions might not be feasible in less specialized centers.

## Conclusions

Our meta-analysis demonstrated that in contrasted to OPD, LPD could achieve short-term advantages within blood loss, and postoperative morbidity for pancreatic-head and periampullary malignancy. Moreover, both procedures have comparable oncological and long-term survival outcomes. LPD may serve as a promising alternative to OPD in selected individuals suffered pancreatic-head and periampullary malignancy. However, taking into account all the limitations of the study, methodologically high-quality controlled clinical trials are necessary for further evaluation. Anyway, we believe our study could serve as a useful background for future researches.

## Abbreviations

CI: Confidence intervals
HR: Hazard ratio
ISGPF: International Study Group for Pancreatic Fistula
LDP: Laparoscopic distal pancreatectomy
LPD: Laparoscopic pancreaticoduodenectomy
MIPD: Minimally invasive pancreaticoduodenectomy
NOS: Newcastle-Ottawa Quality Assessment Scale
NRCT: Nonrandomized comparative study
OPD: Open pancreaticoduodenectomy
OR: Odds ratio
PAAC: Periampullary adenocarcinoma
PD: Pancreaticoduodenectomy
PDAC: Pancreatic duct adenocarcinoma
RCT: Randomized controlled trial
RLN: Retrieved lymph nodes
RPD: Robotic pancreaticoduodenectomy
SD: Standard deviation
SE: Standard error
WMD: Weighted mean difference
